# Insulin Resistance Is a Risk Factor for Overall Cerebral Small Vessel Disease Burden in Old Nondiabetic Healthy Adult Population

**DOI:** 10.3389/fnagi.2019.00127

**Published:** 2019-06-12

**Authors:** Xiaoli Yang, Shufan Zhang, Zhiyuan Dong, Yincui Zi, Yufan Luo, Zhi Jin, Lei Shi, Chen Li, Chuanchen Ren, Danhong Wu

**Affiliations:** ^1^Department of Neurology, The Fifth People’s Hospital of Shanghai, Fudan University, Shanghai, China; ^2^Department of Emergency, The Fifth People’s Hospital of Shanghai, Fudan University, Shanghai, China

**Keywords:** cerebral small vessel disease, total CSVD score, insulin resistance, homeostasis model assessment-estimated insulin resistance index, risk factors

## Abstract

**Background and Purpose**: This study aimed to investigate the association between insulin resistance (IR) and the overall cerebral small vessel disease (CSVD) burden.

**Methods**: We recruited elderly, nondiabetic, healthy subjects prospectively. The overall effect of CSVD on the brain was described by a validated CSVD score. The homeostasis model assessment–estimated insulin resistance index (HOMA-IR) was used for IR estimation, and HOMA-IR ≥2.80 was defined as IR. We evaluated the association between IR and the increasing severity of CSVD score by ordinal regression models adjusting for demographics and cardiovascular risk factors.

**Results**: A total of 156 healthy participants were recruited. The mean age was older in the IR group than in the non-IR group (70.03 vs. 67.45, *p* = 0.04), and the prevalence of hypertension was significantly higher in the IR group than in the non-IR group (82.35% vs. 53.28%, *p* < 0.01). In ordinal regression analysis, IR was positively associated with increasing severity of the total CSVD score (adjusted odds ratio, 3.74; 95% confidence interval, 1.63–5.08; *p* < 0.01) after adjusting traditional risk factors. Furthermore, HOMA-IR levels showed a positive dose-dependent correlation with the total CSVD score (*p* < 0.01, p for trend <0.01).

**Conclusions**: IR is independently associated with increasing severity of the overall CSVD burden, independent of other clinical risk factors in an elderly, nondiabetic, healthy population. Furthermore, HOMA-IR level is correlated with the CSVD burden in a dose-dependent manner.

## Introduction

Cerebral small vessel disease (CSVD) is a syndrome including clinical, neuroimaging, and neuropathological manifestation, caused by various conditions involving perforating vessels (Wardlaw et al., 2013a,b). The pathological mechanism for CSVD is not clear, but more evidence has accumulated for the hypothesis that endothelial dysfunction and atherosclerosis are the primary pathological mechanisms of CSVD. The clinical manifestations of CSVD may be presented as stroke (Sudlow and Warlow, 1997; Debette and Markus, 2010), cognitive impairment (Gorelick et al., 2011), psychiatric disturbance (van Agtmaal et al., 2017), and physical disabilities (Su et al., 2017). Neuroimaging features are diverse, so that the total CSVD score was created to capture the overall effect of CSVD on the brain, which incorporates white matter hyperintensity (WMH), cerebral microbleeds (CMB), lacunes, and enlarged perivascular spaces (EPVS; Huijts et al., 2013; Staals et al., 2014). Although there are some known risk factors for CSVD, such as old age, hypertension and atherosclerosis (Del Brutto and Mera, 2017; Rutten-Jacobs and Markus, 2017), the risk of others is not yet defined.

Insulin resistance (IR) is hypo-reactivity to insulin and is correlated with endothelial dysfunction and atherosclerosis (Ginsberg, 2000; Kernan et al., 2002; Cartolano et al., 2017). Studies suggested that IR increased the incidence and recurrence of stroke (Rundek et al., 2010; Jing et al., 2017), and decreased cortical perfusion (Hoscheidt et al., 2017) and cognitive function in neurologically healthy adults (Huijts et al., 2013; Willette et al., 2013). Furthermore, emerging data demonstrated a possible causal relationship between IR and individual components of CSVD (lacunar infarction; Dearborn et al., 2015; Lee et al., 2016), but little is known about the associations between IR and overall CSVD burden as different neuroimaging components of CSVD shared a common mechanism; therefore, the objective of this study, is to investigate whether IR is associated with the overall CSVD burden, independent of other clinical risk factors in an elderly, nondiabetic, healthy population.

## Materials and Methods

### Study Population

“Investigation on the Status of Cerebrovascular Diseases and Establishing Cohort in Shang Hai Aging Population (ISCDECSHAP)” is a prospective, population-based, and cohort study of stroke incidence and risk factors in an aging population from Shang Hai City. The ISCDECSHAP study aimed to establish a Chinese CSVD community cohort, and was approved by the ethics committee of Hua Shan Hospital and the Fifth People’s Hospital of Shanghai. Written informed consent was obtained from all the patients or their representatives before data collection. Based on protocol, all the subjects, at least 60 years old, performed Cerebral magnetic resonance imaging (MRI), Cerebral MRA, carotid artery ultrasound, cognitive function, and hematologic examination. All the examinations of this study were completed within a week. We only recruited elderly, nondiabetic, healthy subjects from this population into our study. Subjects who met any one of the following criteria were excluded: history of stroke, cognitive dysfunction, heart disease, malignancies, hepatic or renal diseases.

### Clinical and Laboratory Variables

Baseline demographic characteristics including age, sex, smoking status, and comorbid conditions such as hypertension and dyslipidemia were obtained through face-to-face interviews by neurological resident. Fasting venous blood samples were used to measure glucose, insulin, and other laboratory examinations. Intracranial arterial stenosis was defined as more than 50% stenosis in cranial MRA (Adams et al., 1993). Carotid artery stenosis was defined as more than 50% stenosis in carotid artery ultrasound (Adams et al., 1993).

### Brain MRI and the Total CSVD Score

Neuroimaging examinations were performed using a 3.0T MRI system. Axial T2-weighted sequences, fluid-attenuated inversion recovery (FLAIR), T1-weighted sequences, diffusion-weighted imaging (DWI) and axial susceptibility-weighted imaging (SWI) were used for image review. Individual imaging features of CSVD was observed strictly by Neuroimaging standards (Wardlaw et al., 2013a). The Fazekas scale was used for periventricular and deep WMH evaluation (Fazekas et al., 1987). Lacune was numbered and defined as lesions with a diameter of 3 mm-15 mm and a signal similar to CSF, but with a surrounding rim of hyperintensity on FLAIR images. EPVS was distinguished from lacune in size, because EPVS were smaller than 3 mm and had no surrounding rim on FLAIR images; EPVS was numbered just in the basal ganglia and centrum semiovale based on a semi-quantitative scale, which was defined by 1 grade (number is 0–10), 2 grade (number is 11–25), and 3 grade (number >25; Klarenbeek et al., 2013; Xiao et al., 2015). CMB was defined as rounded or ovoid hypointensity foci with, generally, a diameter of 2–5 mm on SWI images (less than 10 mm maximally in diameter).

The total CSVD score was calculated as follows, with 1 point being awarded for each of these features: periventricular Fazekas score =3, or deep Fazekas score ≥2, for WMH; 1 or more lacunes; 1 or more CMBs; and grade 2-3 for EPVS. Therefore, the total CSVD score was stratified from 0 to 4. The MRI were independently evaluated by two neurologists, and inter-observer agreement values for the presence of CMB, WMH, EPVS, and lacune were 0.80, 0.83, 0.78, and 0.79, respectively. Any disagreement regarding the presence of CSVD features was resolved by consensus with the third neuroimaging expert.

### Insulin Resistance Evaluation

IR was evaluated by the homeostatis model assessment–insulin resistance (HOMA-IR), which was calculated as fasting insulin (μU/mL) × fasting glucose (mmol/L)/22.5 (Matthews et al., 1985).

### Statistical Analyses

Statistical analyses were performed using the SPSS21.0 software. For each demographic and clinical feature, normal distribution continuous variables were presented as mean ± standard deviation and compared by an independent sample *t*-test. Abnormal distribution continuous variables were presented as median (interquartile range) and compared by a nonparametric test. Categorical variables were expressed as frequency (percentage) and compared by *χ*^2^ test or Fisher exact test. The association between the total CSVD score and IR was analyzed by ordinal logistic regression.

To assess the dose-dependence of the relationships between HOMA-IR and the total CSVD score, we compared mean HOMA-IR levels with different burdens of each CSVD score by the Kruskal-Wallis test and Jonckheere-Terpstra test. *p* < 0.05 were considered to be statistically significant.

## Results

### Study Population Characteristics

Our study included 156 healthy subjects. The sample size was limited, so we defined IR using HOMA-IR ≥2.80, as this is the same criteria as was used in a study focused on IR among Non-Diabetic Individuals from USA (Rundek et al., 2010). Among the 156 subjects, 34 showed IR and 122 showed non-IR. Subjects with IR differed significantly from those non-IR by age, total cholesterol, fasting glucose, fasting insulin, glycosylated hemoglobin, and prevalence of hypertension (*p* < 0.05), but not by sex, drinking, smoking, CRP, and other cardiovascular risk factors (Table 1).

**Table 1:** Characteristics of subgroups based on insulin resistance (IR).

	non-IR *n* = 122	IR *n* = 34	*p*-value
Age	67.45 ± 5.92	70.03 ± 7.30	0.04
Male	39 (31.97%)	12 (35.29%)	0.72
BMI	23.55 ± 2.72	24.53 ± 2.43	0.06
Drinking	13 (10.66%)	3 (8.82%)	1.00
Smoking	21 (17.21%)	4 (11.76%)	0.60
Hypertension	65 (53.28%)	28 (82.35%)	<0.01
Dyslipidemia	42 (34.43%)	15 (44.12%)	0.30
Total cholesterol (mmol/L)	4.08 ± 1.05	4.53 ± 1.07	0.03
HDL-C (mmol/L)	1.40 ± 0.37	1.25 ± 0.32	0.04
LDL-C (mmol/L)	2.92 (2.32, 3.51)	2.80 (2.03, 3.26)	0.05
Total triglyceride (mmol/L)	1.23 (0.88, 1.63)	1.44 (1.07, 1.74)	0.05
CRP (mg/L)	0.61 (0.28, 1.26)	0.76 (0.42, 2.08)	0.09
Homocysteine (μmol/L)	12 (10.3, 14.2)	12.4 (10.73, 14.83)	0.75
Fasting glucose (mmol/L)	5.12 ± 0.64	6.30 ± 1.34	<0.01
Fasting insulin (μU/mL)	5.67 (2.65, 8.20)	13.19 (11.44, 20.12)	<0.01
Glycosylated hemoglobin	5.60 (5.20, 5.90)	6.0 (5.75, 6.58)	<0.01
Carotid artery stenosis	8 (6.56%)	1 (2.94%)	0.69
Intracranial arterial stenosis	4 (3.28%	4 (11.76%)	0.05

For individual imaging features of CSVD, the distribution of lacunar, CMB and EPVS grade had significant differences between the two groups (Table 2). The proportion of lacunar, CMB and moderate to severe EPVS (EPVS grade ≥2) was much higher in the IR group, but there was no difference in WMH between the two groups, no matter periventricular WMH nor deep WMH. For the severity of overall CSVD burden, 64.71% IR subjects had a CSVD score of more than 1, whereas it was 39.35% for non-IR subjects (Table 2).

**Table 2:** The distribution of neuroimaging features of cerebral small vessel disease (CSVD) based on IR.

	non-IR *n* = 122	IR *n* = 34	*p*-value
Lacunar	29 (23.77%)	19 (55.88%)	<0.01
CMB	27 (22.13%)	14 (41.18%)	0.03
Periventricular WMH			0.80
1	20 (16.39%)	7 (20.59%)	
2	82 (67.21%)	21 (61.76%)	
3	19 (15.57%)	5 (14.70%)	
Deep WMH			0.19
1	39 (31.97%)	11 (32.35%)	
2	59 (48.36%	20 (58.82%)	
3	23 (18.85%)	3 (8.82%)	
EPVS grade			<0.01
1	88 (72.13%)	8 (23.52%)	
2	24 (19.67%)	13 (38.24%)	
3	10 (8.20%)	13 (38.24%)	
CSVD score			<0.01
0	27 (22.13%)	2 (5.88%)	
1	47 (38.52%)	10 (29.41%)	
2	38 (31.15%)	12 (35.30%)	
≥3	10 (8.20%)	10 (29.41%)	

We assessed the association between IR and other cardiovascular risk factors with increasing severity of the total CSVD score by ordered logistic regression. In this statistical model the odds ratio was presumed for each level (Parallel line examination = 0.30) and IR was positively associated with increasing severity of the total CSVD score with OR 3.74 (95% CI was 1.63–5.08, and *p* < 0.01), after adjustment for age, hypertension, and other traditional risk factors (Table 3).

**Table 3:** Association of IR and other cardiovascular risk factors with increasing severity of the total CSVD score.

	OR value	95% CI		*p*-value
Age	1.01	0.95	2.58	0.84
Male	0.71	0.28	1.32	0.46
BMI	1.06	0.94	2.55	0.35
Smoking	1.49	0.48	1.62	0.49
Drinking	1.23	0.36	1.43	0.74
Hypertension	1.49	0.73	2.08	0.27
Hyperlipidemia	0.86	0.41	1.51	0.68
Homocysteine	0.99	0.94	2.55	0.70
Total cholesterol	1.19	0.80	2.22	0.39
Total triglyceride	0.94	0.61	1.84	0.78
HDL-C	0.35	0.12	1.13	0.05
LDL-C	0.95	0.79	2.20	0.58
CRP	1.03	0.98	2.65	0.28
Carotid artery stenosis	0.78	0.18	1.19	0.75
Intracranial arterial stenosis	1.60	0.33	1.39	0.56
IR	3.74	1.63	5.08	<0.01

**Figure 1:**
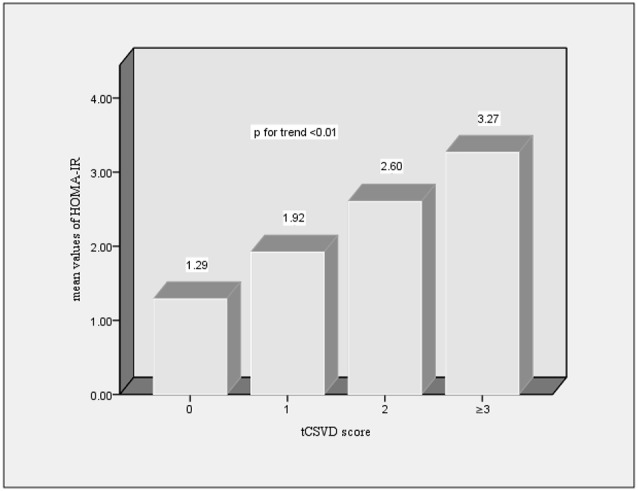
Distribution of mean values of homeostatis model assessment-insulin resistance (HOMA-IR) according to the total cerebral small vessel disease (CSVD) burden of brain.

We investigated the relationship between the HOMA-IR levels and the total CSVD burden further, which revealed a positive dose-dependent correlation between HOMA-IR level and the total CSVD score (*p* < 0.01, p for trend <0.01). Participants who had lower HOMA-IR level displayed lower CSVD score, as suggested in Figure 1.

## Discussion

In this study, we investigated the association between IR and overall CSVD burden in an elderly, nondiabetic, healthy population. After adjustment for established CSVD-related risk factors, we confirmed that IR was independently associated with an increasing severity of overall CSVD burden in multivariate analysis. Furthermore, HOMA-IR level was related with CSVD burden in a dose-dependent manner.

IR is not exclusive to diabetes mellitus but is a core characteristic of a variety of metabolic disorders (Ginsberg, 2000; Kernan et al., 2002) such as hypertension, dyslipidemia, and obesity. In our study, compared with non-IR groups, the IR group had a higher prevalence of hypertension, higher levels of total cholesterol, fasting glucose and fasting insulin, and more advanced age, which all further confirmed that IR is closely associated with metabolic syndrome. Emerging studies focused on CSVD indicated that IR was correlated to cortical perfusion decline (Hoscheidt et al., 2017) and cognitive impairment in healthy adults (Huijts et al., 2013; Willette et al., 2013). Some studies implied there was a possible causal relationship between IR and lacunar infarction, which is a CSVD neuroimaging features (Dearborn et al., 2015; Lee et al., 2016), but research on the association of IR and overall CSVD burden is infrequent. In our study, we found that the IR group had more incident of lacunar, CMB, and EPVS. Furthermore, IR was positively associated with increasing severity of the total CSVD score with OR 3.74 (95% CI was 1.63–5.08, and *p* < 0.01) after adjustment for age, hypertension, and other traditional risk factors, suggesting that IR was a risk factor of overall CSVD burden, irrespective of other known risk factors. Furthermore, the relationship between HOMA-IR level and the total CSVD score presented a positive dose-dependent (*p* < 0.01, p for trend < 0.01). To understand the close relationship, we proposed two possible explanations as follow.

One explanation for our observation is that IR is correlated with endothelial dysfunction. Previous studies demonstrated that insulin can regulate the vascular homeostasis by phosphatidylinositol 3-kinase signaling pathways in endothelium (Muniyappa and Quon, 2007; Muniyappa et al., 2007). IR is hypo-reactivity to insulin and characterized by pathway-specific impairment of phosphatidylinositol 3-kinase signaling pathways. Study suggested that IR accelerated lipolysis and increased the level of inflammatory mediators (Scalia, 2013), all of which may contribute to endothelial dysfunction (Yudkin et al., 2005; Muniyappa and Quon, 2007; Muniyappa et al., 2007; Scalia, 2013). Endothelial dysfunction may contribute to the blood-brain barrier (BBB) disturbance, an increase of the permeability of the BBB, leakage of protease, immunoglobulin, complement components, and cytokines into vessel wall and, subsequently, perivascular tissue (Doubal et al., 2010; Wardlaw, 2010). Eventually, this may initiate or worsen the development of CSVD.

Another explanation for our observation is that IR is associated with atherosclerosis (Cartolano et al., 2017). IR may increase the levels of inflammatory mediators by changing the secretion of adipokines (Scalia, 2013), and induce chronic and continuous neuroinflammation state subsequently. On the one hand, the neuroinflammation can provide ground for CSVD (Sandu et al., 2015), on the other hand, inflammation involved in atherosclerosis (Hansson, 2017). Furthermore, IR may promote atherosclerosis through disturbing some proatherogenic signaling pathways, such as phosphatidylinositol 3-kinase signaling pathways and mitogen-activated protein kinase signaling pathway. Thus, IR may aggravate a parent artery or perforate arteriole atherosclerosis, contributing to the development of CSVD.

We also investigated the association of age and hypertension with increasing severity of the total CSVD burden. Although age and hypertension were established risk factors for CSVD (Del Brutto and Mera, 2017; Rutten-Jacobs and Markus, 2017), Julie Staals’s study demonstrated that they were significantly and independently associated with the total CSVD score (Staals et al., 2014). However, in our study, we did not find the statistical differences (*p* > 0.05) in multivariate analysis, which agreed with Wiseman’s study (Wiseman et al., 2016). The controversial outcomes may be attributable to the two points. First is the strength of correlation was different between different CSVD neuroimaging features (Wiseman et al., 2016; Del Brutto and Mera, 2017; Rutten-Jacobs and Markus, 2017) while the CSVD scale is a semi-quantitative way to capture the overall effect of CSVD neuroimaging features on the brain, it cannot represent the overall effect of CSVD in neuropathological manifestation. Second is that the characteristics of the participants between studies were different. For instance, our study recruited an elderly, nondiabetic, healthy population from the community, whereas, Julie Staals’ study focused on patients with a definite diagnosis of clinical lacunar or mild cortical ischemic stroke which had been presented to the hospital.

Our study had several limitations. First, as a cross-sectional study, we cannot avoid the selection bias, and the causal relationship between IR and overall CSVD burden cannot be drawn. Second, we estimated IR by the HOMA-IR index, not by the gold standard for IR measurement. However, HOMA-IR is commonly used as an alternative method in clinical and epidemiological studies (Klarenbeek et al., 2013; Willette et al., 2013; Dearborn et al., 2015). Overall, we confirmed that IR was independently associated with the severity of overall CSVD burden and correlated with it in a dose-dependent manner, which implied that IR may be indirectly involved in CSVD by endothelial dysfunction and atherosclerosis in pathologies process. Nevertheless, further study should follow this in the future.

## Data Availability

The raw data supporting the conclusions of this manuscript will be made available by the authors, without undue reservation, to any qualified researcher.

## Ethics Statement

This study was performed according to the principles of the Declaration of Helsinki, and was approved by the ethics committee of Hua Shan Hospital and the Fifth People’s Hospital of Shanghai. Written informed consent was obtained from all individual participants included in the study.

## Author Contributions

DW proposed research design. XY and SZ analyzed data and wrote the article. XY and ZJ evaluated MRI, disagreement was resolved by consensus with DW. ZD, YZ, YL, LS and CL were responsible for collecting data. DW and CR proofread and reviewed the manuscript. ZD polished the final manuscript.

## Conflict of Interest Statement

The authors declare that the research was conducted in the absence of any commercial or financial relationships that could be construed as a potential conflict of interest.
